# Mixed‐meal tolerance test to assess residual beta‐cell secretion: Beyond the area‐under‐curve of plasma C‐peptide concentration

**DOI:** 10.1111/pedi.12816

**Published:** 2019-02-19

**Authors:** Yue Ruan, Ruben H. Willemsen, Malgorzata E. Wilinska, Martin Tauschmann, David B. Dunger, Roman Hovorka

**Affiliations:** ^1^ Wellcome Trust‐MRC Institute of Metabolic Science University of Cambridge Cambridge UK; ^2^ Oxford Centre for Diabetes, Endocrinology and Metabolism University of Oxford Oxford UK; ^3^ Department of Paediatrics University of Cambridge Cambridge UK

**Keywords:** beta‐cell secretion, C‐peptide, mixed‐meal tolerance test, type 1 diabetes

## Abstract

**Aims:**

Residual beta‐cell secretion in type 1 diabetes is commonly assessed by area‐under‐curve of plasma C‐peptide concentration (AUC_Cpep_) following mixed‐meal tolerance test (MMTT). We aimed to investigate alternative measures of beta‐cell responsiveness.

**Methods:**

We analyzed data from 32 youth (age 7 to 17 years) undergoing MMTT within 6 months of type 1 diabetes diagnosis. We related AUC_Cpep_ with (a) validated mechanistic index of postprandial beta‐cell responsiveness *M*_I_ accounting for glucose level during MMTT, and (b) pragmatic marker calculated as baseline plasma C‐peptide concentration corrected for baseline plasma glucose concentration.

**Results:**

Postprandial responsiveness *M*_I_ was correlated with age and BMI SDS (*R*_*s*_ = 0.66 and 0.44, *P* < 0.01 and *P* < 0.05) and was more correlated with glycated hemoglobin than AUC_Cpep_ (*R*_*s*_ = 0.79, *P* = 0.04). The pragmatic marker was highly correlated with AUC_Cpep_ (*R*_*s*_ = 0.94, *P* < 0.01).

**Conclusions:**

Postprandial responsiveness *M*_I_ may be more relevant to glucose control than AUC_Cpep_. Baseline C‐peptide corrected for baseline glucose appears to be a suitable surrogate of AUC_Cpep_ if MMTT is not performed.

## 1 INTRODUCTION

The area‐under‐curve of sequential C‐peptide concentrations (AUC_Cpep_) during the mixed‐meal tolerance test (MMTT) is the gold‐standard method to assess residual beta‐cell (ie, insulin) secretion in type 1 diabetes.^1^ Traditionally, glucose excursions during the MMTT are not taken into account, although these impact on the magnitude of C‐peptide response.^2^

In this work, we re‐analyzed MMTT data obtained in newly diagnosed children and adolescents with type 1 diabetes aged 7 to 17 years^2^ (a) to identify surrogate mechanistic and pragmatic markers of AUC_Cpep_ and (b) to explore the relationships among demographic and clinical factors, and AUC_Cpep_ and its surrogate markers.

## 2 METHODS

We analyzed data obtained from 32 participants with newly diagnosed type 1 diabetes (age 12.4[2.9] years, 12 males, HbA1c 6.8%[1.1], BMI SDS 0.62[1.02], total daily dose of insulin 0.57[0.23] U/kg; mean [SD]) who underwent MMTT within 6 months of diagnosis (mean time since diagnosis 142[38] days).^2^ The National Research Ethics Committee East of England‐Cambridge South approved the study.

All participants (aged ≥16 years) gave informed consent, and children <16 years gave assent and their parents gave informed consent to the study procedures.

The MMTT was performed following an overnight fast, with no food or drink other than water from midnight, and at baseline glucose levels between 4 and 11.1 mmol/L. Long‐acting insulin and basal rates for insulin pump users were continued as normal. The use of rapid‐acting insulin bolus was acceptable up to 2 hours before the MMTT and the use of short‐acting insulin bolus up to 6 hours before the MMTT. Participants ingested 6 mL/kg of Boost meal solution (maximum 360 mL), within 10 minutes. Blood samples for the measurement of C‐peptide and glucose were collected 10 minutes prior to the meal (−10 minutes), at the time of ingestion (0 minutes), and at 15, 30, 60, 90 and 120 minutes.

Plasma C‐peptide was assayed in singleton on a Diasorin Liaison XL automated immunoassay analyzer using a one‐step chemiluminescence immunoassay (Diasorin S.p.A, 13040 Saluggia [VC], Italy). Glucose levels were analyzed via an adaption of the hexokinase‐glucose‐6‐phosphate dehydrogenase method.^3^ HbA1c was analyzed on the Tosoh G8 High Performance Liquid Chromatography Analyzer using the gold standard ion‐exchange method with <2% between‐batch imprecision (Tosoh Bioscience, Inc., South San Francisco, CA).

AUC_Cpep_ and incremental IAUC_Cpep_ was calculated using the trapezoidal method. A compartment model validated in normal subjects and those with newly diagnosed type 2 diabetes^4^ of C‐peptide kinetics was used to estimate two mechanistic indices of pancreatic beta‐cell responsiveness: (a) postprandial responsiveness *M*_*I*_ (ability of postprandial glucose to stimulate beta‐cell insulin secretion; a change in plasma glucose by 1 mmoL/L results in a change in the C‐peptide secretion by *M*_*I*_ pmol/L/min) and (b) basal responsiveness *M*_0_ (ability of fasting glucose to stimulate beta‐cell insulin secretion; *M*_0_ approximates fasting C‐peptide divided by the fasting plasma glucose concentration) (see Supporting Information, Appendix S1 ).^4^ Baseline plasma C‐peptide concentration divided by the baseline plasma glucose concentration was calculated as a pragmatic marker of beta‐cell function.

The Spearman rank correlation coefficient was used to explore relationships between age, BMI SDS (body mass index SD score), HbA1c, total daily dose of insulin, baseline plasma C‐peptide, AUC_C__pep_, baseline plasma C‐peptide divided by baseline plasma glucose, IAUC_Cpep_, insulin dose‐adjusted A1C (IDAA1C) defined as A1C (percent) + [4 × insulin dose (units per kilogram per 24 hours)],^5^ *M*_0_ and *M*_I_. Fisher's r‐to‐z transformation was applied for testing the difference between two Spearman rank correlation coefficients.^6^ *P* values less than 0.05 were considered statistically significant. As these are post hoc evaluations, all observations are considered exploratory. Statistical analyses were performed using SPSS, version 21 (IBM Software, Hampshire, UK). Data are reported as mean (SD) or median [interquartile range], unless stated otherwise.

## 3 RESULTS

The postprandial and fasting beta‐cell responsiveness *M*_I_ and *M*_0_ were estimated at 3.3[1.6‐5.4] and at 3.1[2.0‐4.5] 10^−9^/minutes, respectively. Figure S1 shows a sample model fit to measured plasma C‐peptide including measurements of plasma glucose (the forcing function). Figure S2 depicts weighted residuals across all participants demonstrating acceptable fit of the model to plasma C‐peptide measurements.

Table 1 reports the Spearman rank correlation among demographic and clinical factors, AUC_Cpep_ and its surrogate markers. The strongest correlations found for age and BMI SDS were with *M*_I_ (R_S_ = 0.66 and 0.44, respectively, *P* < 0.01 and *P* < 0.05, respectively). The total daily dose of insulin was inversely correlated with *M*_0_ (*R*_*S*_ = −0.42, *P* < 0.05) and baseline C‐peptide over baseline glucose (*R*_*S*_ = −0.38, *P* < 0.05).

**Table 1:** Spearman rank correlation between demographic/clinical factors and markers of beta‐cell responsiveness (N = 32)

	Age (y)	HbA1c (%)	BMI SDS	TDD (U/kg)	AUC_Cpep_ (pmol/L/min)	Baseline C‐peptide (pmol/L)	Baseline C‐peptide over baseline glucose	*M*_0_ (/min)	*M*_I_ (/min)	IDAA1c	IAUC_Cpep_ (pmol/L/min)
Age (y)	1.00	−0.34	0.14	−0.08	**0.46** **	0.27	0.31	0.27	**0.66** **	−0.31	0.50
HbA1c (%)		1.00	0.01	−0.01	−0.19	−0.08	−0.13	−0.15	**−0.36** *	**0.72** **	−0.14
BMI SDS			1.00	−0.01	**0.41** *	**0.38** *	**0.41** *	**0.36** *	**0.44** *	−0.18	**0.69** **
TDD (U/kg)				1.00	−0.32	−0.27	**−0.38** *	**−0.42** *	−0.28	**0.64** **	−0.22
AUC_Cpep_ (pmol/L/min)					1.00	**0.88** **	**0.94** **	**0.92** **	**0.79** **	−0.36	**0.99** **
Baseline C‐peptide (pmol/L)						1.00	**0.95** **	**0.89** **	**0.54** **	−0.24	**0.87** **
Baseline C‐peptide over baseline glucose							1.00	**0.94** **	**0.67** **	−0.35	**0.91** **
*M*_0_ (/min)								1.00	**0.63** **	−0.37	**0.89** **
*M*_I_ (/min)									1.00	−0.48	**0.79** **
IDAA1c										1.00	−0.28
IAUC_Cpep_ (pmol/L/min)											1.00

* *P* < 0.05,

** *P* < 0.01. Significant correlations are shown in boldface.

Abbreviations: BMI SDS, body mass index SD score; IDAA1c, insulin‐dose adjusted HbA1c; IAUC_Cpep_, incremental area‐under‐curve of C‐peptide; TDD, total daily dose of insulin.

**Figure 1:**
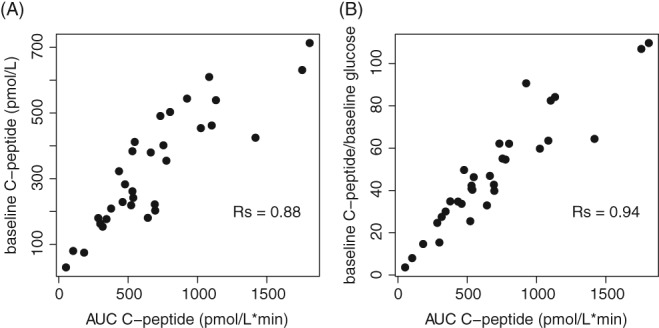
The scatter plot of AUC_Cpep_ vs baseline C‐peptide (A) and AUC_Cpep_ vs baseline C‐peptide corrected for baseline glucose level (B)

Figure 1 demonstrates that AUC_Cpep_ has a stronger correlation with baseline C‐peptide corrected for baseline glucose (*R*_*S*_ = 0.94) than baseline C‐peptide per se (*R*_*S*_ = 0.88). Figure S3 relates baseline HbA1c vs AUC_Cpep_ and log‐transformed *M*_I_. AUC_Cpep_ was not correlated with HbA1c (*R*_*S*_ = −0.19, *P* = NS) whereas *M*_I_ is (*R*_*S*_ = −0.36, *P* < 0.05); the difference between the two correlation coefficients is statistically significant (*P* = 0.04).

## 4 DISCUSSION

The present analysis demonstrates the feasibility of using a model of C‐peptide kinetics to assess residual beta‐cell function during MMTT in newly‐diagnosed type 1 diabetes. Traditionally, the AUC_Cpep_ during a MMTT has not been corrected for glucose excursions, which are likely to affect the amplitude of the C‐peptide response.^2^ Our data show that using more advanced measures of beta‐cell function, such as *M*_*I*_ and *M*_*0*_, can identify meaningful correlations with clinical parameters such as TDD and HbA1c, which are not identified using uncorrected AUC_Cpep_. In addition, we show baseline C‐peptide corrected for baseline glucose to be a surrogate marker of AUC_Cpep_.

The basal responsiveness *M*_*0*_ and the postprandial responsiveness *M*_*I*_ were estimated at median 3.3 and median 3.1 10^−9^/min, respectively. These values are considerably smaller than those estimated in normal subjects where *M*_*0*_ were estimated at a mean of 10.3 and *M*_*I*_ at 90.0 10^−9^/min.^7^ In two subjects, *M*_*I*_ was estimated at zero and in one subject *M*_*0*_ at zero due to the lack of increased C‐peptide levels post‐meal and undetectable C‐peptide level at baseline. These estimations are clinically meaningful as individuals with complete basal and postprandial insulin responsiveness can be identified.

The positive correlations between age and *M*_*I*_, and between BMI SDS and *M*_*I*_ suggest that postprandial responsiveness is more preserved in older and heavier children and adolescents with newly‐diagnosed type 1 diabetes than the younger and lighter individuals.

Figure 1 demonstrates that baseline C‐peptide corrected for baseline glucose is highly correlated with AUC_Cpep_ and could be used as a surrogate marker of insulin secretion instead of AUC_Cpep_. A previous study has shown the plausibility of using 90‐min‐stimulated C‐peptide concentration or baseline C‐peptide as a substitute for AUC_Cpep_ to represent insulin secretion with a similar correlation coefficient R_S_ = 0.96 but in a larger population (N = 421).^8^ Data from the present analysis suggest that baseline C‐peptide corrected for baseline glucose may be a more appropriate marker than baseline C‐peptide and a more cost‐effective marker than the stimulated C‐peptide concentration sidestepping the need for MMTT and complexity of the assessment.

We show that *M*_I_ was more tightly correlated with HbA1c than AUC_Cpep_ (*P* = 0.04) indicating that *M*_I_ may be a more clinically relevant marker of C‐peptide secretion than AUC_Cpep_. The study is limited by a relatively small sample size. Further analyses with larger datasets and longitudinal evaluations are warranted. We applied parameters of C‐peptide kinetics determined in healthy subjects. As C‐peptide is eliminated primarily by the kidney and assuming comparable kidney function among healthy individuals and those with recently diagnosed type 1 diabetes, we consider this limitation to be of little significance to our findings.

Alternative C‐peptide secretion models assume a more complex relationship between glucose concentration and insulin secretion compared to the model used in the present study.^9^, ^10^ These alternative models may provide additional information about C‐peptide secretory characteristics but require more frequent sampling. Our parameter *M*_I_ represents the dominant relationship between plasma glucose and C‐peptide secretion and is accounted for in the alternative approaches.^9^, ^10^

In conclusion, baseline C‐peptide corrected for baseline glucose may be a suitable surrogate marker of residual beta‐cell in newly‐diagnosed type 1 diabetes. Postprandial pancreatic responsiveness estimated through a model of C‐peptide kinetics appears more relevant to glucose control than the conventional area‐under‐curve of plasma C‐peptide concentration following MMTT.

## CONFLICTS OF INTEREST

R.H. reports having received speaker honoraria from Eli Lilly and Novo Nordisk, serving on advisory panel for Eli Lilly and Novo Nordisk, receiving license fees from BBraun and Medtronic; and having served as a consultant to BBraun. M.E.W. has received license fees from Becton Dickinson and has served as a consultant to Beckton Dickinson. Y.R., M.T., R.H.W. and D.B.D. declare no competing financial interests exist.

## Supporting information


**Appendix S1**. Model of C‐peptide kinetics during mixed‐meal tolerance test.
**Figure S1.** A sample model fit from a participant undergoing MMTT (meal ingested at time 0 minutes). The green solid line with circles represents the measured plasma C‐peptide; the blue dashed line with triangles represents model fit; the red solid line with crosses represents the measured plasma glucose (the forcing function).
**Figure S2**. Normalized residuals (weighted by the measurement error with 6% coefficient of variation) of model fit to the plasma C‐peptide concentration (mean ± SD, N = 32).
**Figure S3**. Scatter plot of baseline HbA1c vs AUC_Cpep_ (left panel) and scatter plot of baseline HbA1c vs log‐transformed postprandial pancreatic responsiveness M_I_ (right panel) (*R*_*S*_, Spearman correlation coefficient).
