# Supplementary material for: Mixed‐meal tolerance test to assess residual beta‐cell secretion: Beyond the area‐under‐curve of plasma C‐peptide concentration
Source: Pediatr Diabetes. 2019 Feb 19;20(3):282–5. doi: 10.1111/pedi.12816 (PMC6487945; doi:10.1111/pedi.12816)
Supplement: Supplementary file 1 — Appendix S1. Model of C‐peptide kinetics during mixed‐meal tolerance test. Figure S1. A sample model fit from a participant undergoing MMTT (meal ingested at time 0 minutes). The green solid line with circles represents the measured plasma C‐peptide; the blue dashed line with triangles represents model fit; the red solid line with crosses represents the measured plasma glucose (the forcing function). Figure S2. Normalized residuals (weighted by the measurement error with 6% coefficient of variation) of model fit to the plasma C‐peptide concentration (mean ± SD, N = 32). Figure S3. Scatter plot of baseline HbA1c vs AUCCpep (left panel) and scatter plot of baseline HbA1c vs log‐transformed postprandial pancreatic responsiveness MI (right panel) (R S, Spearman correlation coefficient). [file PEDI-20-282-s001.docx]

**Supplemental Material**


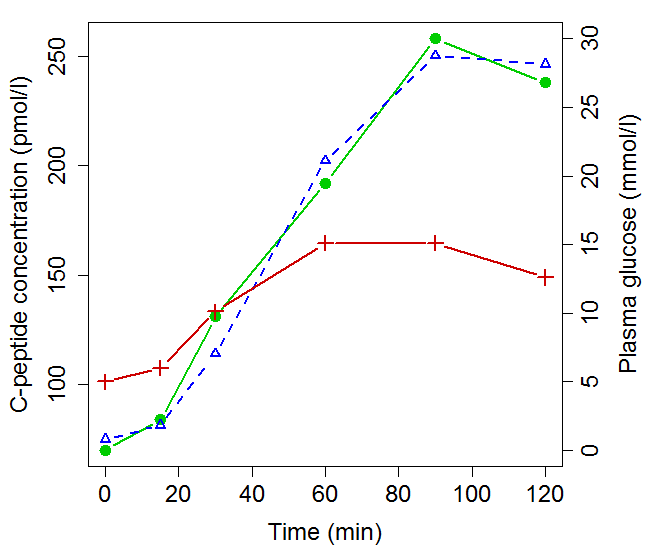


Figure S1. A sample model fit from a participant undergoing MMTT (meal ingested at time 0min). The green solid line with circles represents the measured plasma C-peptide; the blue dashed line with triangles represents model fit; the red solid line with crosses represents the measured plasma glucose (the forcing function).


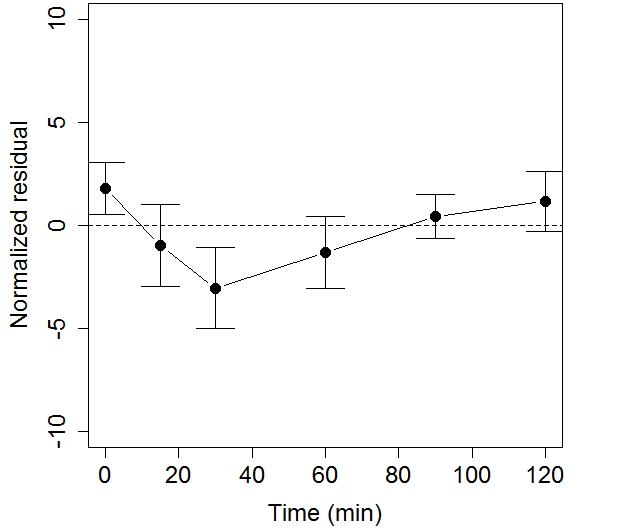


Figure S2. Normalized residuals (weighted by the measurement error with 6% coefficient of variation) of model fit to the plasma C-peptide concentration (mean ± standard deviation, N =32).


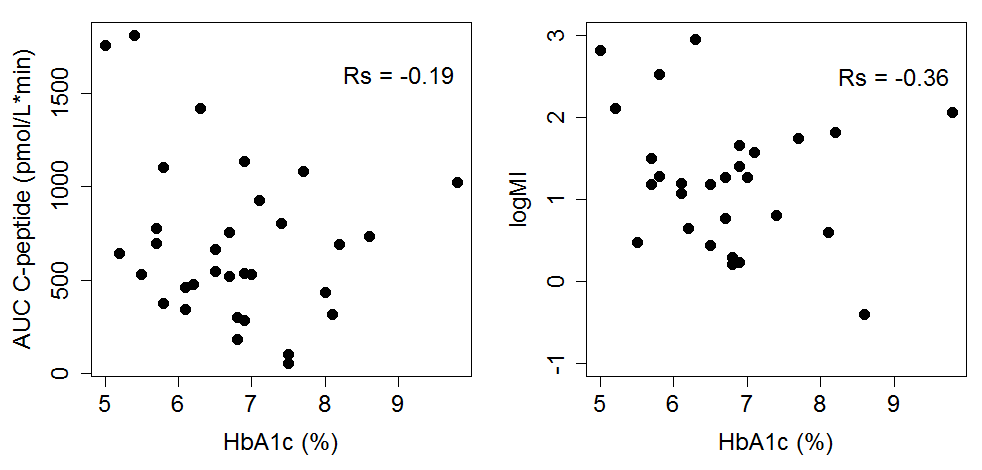


Figure S3. Scatter plot of baseline HbA1c vs. AUC_Cpep_ (left panel) and scatter plot of baseline HbA1c vs. log-transformed postprandial pancreatic responsiveness M_I_ (right panel) (R_S_: Spearman correlation coefficient).

**Model of C-peptide kinetics during mixed-meal tolerance test**

The model assumes that C-peptide secretion is linearly related to the blood glucose concentration ([1](#_ENREF_1)). The linear relationship is imposed from the time ingestion of the mixed meal until the last blood sample taken or when the plasma C-peptide concentration decreases below the baseline (pre-meal) C-peptide concentration ([1](#_ENREF_1)). The model of C-peptide kinetics is described by the following set of differential equations:

$$\begin{matrix} \frac{dc_{1}(t)}{dt}=-(k_{01}+k_{21})c_{1}(t)+k_{12}c_{2}(t)+u(t)\text{ }c_{1}(0)=u(0)/k_{01} \\ \frac{dc_{2}(t)}{dt}=k_{21}c_{1}(t)-k_{12}c_{2}(t)\text{ }c_{2}(0)=c_{1}(0)k_{21}/k_{12} \\ u(t)=\left\{\begin{matrix} M_{I}(g(t)-g_{b})+M_{0}g_{b}\text{ if }M_{I}(g(t)-g_{b})+M_{0}g_{b}>0, \\ 0\text{ otherwise, }0\leq t\leq t_{\max} \end{matrix}\right\} \end{matrix}$$

where $c_{1}(t)$ is C-peptide concentration in the central (plasma) compartment (nmol/l), $c_{2}(t)$ is equivalent concentration in the peripheral compartment (nmol/l), *i.e.* the amount of C-peptide in the peripheral compartment per unit volume of the central compartment, $k_{ij}$ are transfer rate constants (per min), $g(t)$ is plasma glucose concentration (mmol/l), $g_{b}$ is fasting plasma glucose concentration (mmol/l), $u(t)$ is the secretion rate of C-peptide per unit volume of the central compartment and is constrained to nonnegative values (nmol/l/min), $M_{I}$,$M_{I}\geq0$, is the postprandial sensitivity index (/min), $M_{0}$,$M_{0}\geq0$, is the basal sensitivity index (/min), and *t_max_* is 120 min. $M_{I}$represents the ability of postprandial glucose to stimulate beta-cell. A change in plasma glucose by 1 mmol/l results in a change in the C-peptide secretion by $M_{I}$pmol/l/min. $M_{0}$represents the ability of fasting glucose to stimulate beta-cell. $M_{0}$is numerically equal to the fasting C-peptide divided by the fasting plasma glucose concentration. Both $M_{I}$ and $M_{0}$ are normalized to the distribution volume of C-peptide in the central compartment.

The population-based estimates of C-peptide kinetics were used to obtain transfer rate constants $k_{ij}$from the subject’s age and classification (‘normal’ classification was used for all the 32 subjects studied in the present analysis assuming similar transfer rate constants between normal subjects and type 1 diabetes [other categories are ‘obese’ and ‘type 2 diabetes’([2](#_ENREF_2))]). The model input is the plasma glucose concentration (the forcing function) and the model output is the plasma C-peptide concentration. The model parameters are $M_{I}$ and $M_{0}$ estimated employing the weighted nonlinear regression analysis implemented in the program CPR Version 1.0 (CPR: Calculating Pancreatic Responsiveness, Subasinghe, H. and Hovorka, R., City University, London). The measurement errors of the plasma C‑peptide concentration were assumed to be uncorrelated, with zero mean and a constant coefficient of variation of 6% comparable to the assay error.

**References**

1. Hovorka R, Chassin L, Luzio SD, Playle R, Owens DR: Pancreatic beta-cell responsiveness during meal tolerance test: model assessment in normal subjects and subjects with newly diagnosed noninsulin-dependent diabetes mellitus. J Clin Endocrinol Metab 1998;83:744-750

2. Van Cauter E, Mestrez F, Sturis J, Polonsky KS: Estimation of insulin secretion rates from C-peptide levels. Comparison of individual and standard kinetic parameters for C-peptide clearance. Diabetes 1992;41:368-377
